# Unveiling the black box: imperative for explainable AI in cardiovascular disease prevention

**DOI:** 10.1016/j.lanwpc.2024.101145

**Published:** 2024-07-13

**Authors:** Yanyi Wu, Chenghua Lin

**Affiliations:** a School of Public Affairs, Zhejiang University, Hangzhou 310058, China; b Institute of China’s Science, Technology and Policy, Zhejiang University, Hangzhou 310058, China

Dalakoti et al. have a positive attitude towards the application of artificial intelligence (AI) in cardiovascular disease (CVD) prevention in Singapore, especially emphasizing the potential of AI tools such as CardioSight and CHAMP in identifying high-risk individuals and implementing preventive measures.^1^ However, a core issue is missing from its argument: the explainability and transparency of these AI systems. Given the increasing penetration of AI technology into medical decision-making, it has become a top priority to uncover the mystery of the “black box” and ensure that the recommendations provided by these systems can be understood and trusted by both physicians and patients alike.

The lack of explainability in AI systems poses significant challenges in the medical domain, because medical decisions directly impact patient health and safety. Physicians need to comprehend the logical basis of AI recommendations to communicate effectively with patients and make prudent decisions.^2^ For example, when CardioSight identifies that a patient is at high risk for CVD, the physician must be able to understand and explain the factors contributing to this assessment, otherwise, they may miss the early intervention opportunities because of the uncertainty of AI recommendations. In parallel, patients have the right to know how their health data is being used and how AI algorithms arrive at specific conclusions. The opaque systems will erode patients' trust, hinder patient-physician communication, and impede shared decision-making.^3^ Must be aware that the success of AI-driven CVD prevention depends not only on the accuracy of predictions but also on the transparency of the decision-making process.

Therefore, researchers and developers should give priority to the development of explainable AI models in healthcare, which involves designing algorithms that can provide clear, interpretable explanations for their predictions and recommendations.^4^ Techniques such as feature importance analysis, rule extraction and counterfactual explanation to unravel the complex process of AI decision-making.^5^ Increasing investment in explainable AI aims to foster trust, facilitate effective communication, and ensure that AI tools such as CardioSight and CHAMP are powerful, transparent and accountable for realizing the fundamental change of cardiovascular disease prevention and patient care model.

## Declaration of interests

Authors declare no competing interests.
